# A tailored within-community specimen collection strategy increased uptake of cervical cancer screening in a cross-sectional study in Ghana

**DOI:** 10.1186/s12889-017-4631-y

**Published:** 2017-08-01

**Authors:** Adolf K. Awua, Edwin K. Wiredu, Edwin A. Afari, Ahmad S. Tijani, Gabriel Djanmah, Richard M. K. Adanu

**Affiliations:** 1 Department of Epidemiology and Disease Control, School of Public Health, University of Ghana, Accra, Ghana; 2 Cellular and Clinical Research Centre, Radiological and Medical Sciences Research Institute, GAEC, Accra, Ghana; 3 Population, Family and Reproductive Health, School of Public Health, University of Ghana, Accra, Ghana; 4 University of Health and Allied Sciences, Ho, Ghana; 5 Department of Pathology, School of Biomedical and Allied Health Science, College of Health Sciences, University of Ghana, Korle-Bu, Accra, Ghana; 6 Akuse Government Hospital, Ghana Health Service, Akuse, Ghana

**Keywords:** Cervical cancer, Cervical screening, Self-sampling, HPV testing, Pap smear, Ghana

## Abstract

**Background:**

The implementation of cervical cancer screening strategies has reported different rates of success in different countries due to population specific factors that limit women’s participation. We report observations and the development of a community-based specimen collection strategy which resulted from interactions with women in the study communities, following an initial low response to a hospital based cervical cancer screening strategy.

**Method:**

Women were recruited by a house survey and invited to report at a hospital either within a week or after a week for self and health-personnel specimen collections. However, due to the very low response and subsequent interactions with the women of the communities, another strategy was developed that required recruited women report at a central location within their respective communities for specimen collections at times that did not interfere with their daily routines.

**Results:**

For specimen collection, of the 156 participants who opted to report after a week at the hospital, 60 (38.5%) reported. Of the 118 participants who opted to report within 1 week at the hospital, 55 (46.6%) reported. Of the 103 participants were invited to report at a specified location within the community, 98 (95.1%) reported. An overall response rate of 60.4% was attained. Almost 89.7% (226 of 253) of the women performed both self and health personnel sample collection.

**Conclusion:**

The community-based strategy with self-specimen collection and HPV testing holds great potential for increasing women’s participation in cervical cancer screening in Ghana as compared to the hospital based strategy.

**Electronic supplementary material:**

The online version of this article (doi:10.1186/s12889-017-4631-y) contains supplementary material, which is available to authorized users.

## Background

The global burden of cervical cancer, which was estimated in the year 2012 to included 527,624 incident cases, age-standardized incidence rate (ASR) of 14.0 per 100,000 women per year, and 265,653 deaths undoubtedly makes cervical cancer a significant global health concern [1]. The greatest proportion of this global burden of cervical cancer (84.3%) were estimated to have occurred in developing countries particularly in the sub-Sahara Africa region. The estimated burden in Ghana, 3062 incident cases per year, [1–3] indicates cervical cancer is a significant public health issue that requires attention. The implementation of high quality screening programs with hospital-based Pap smear test has been credited with the drastic reductions (> 75%) in both incidence and mortality due to cervical cancer in developed countries [4–6]. However, the current rates of cervical cancer screening coverage in some developed countries still need to be improved [7]. Moreover, reductions in cervical cancer rates are yet to be achieved in developing countries, even with the hospital-based Pap smears testing; This has been due to the low quality of implementation, difficulties in attaining the requirements of the Pap smear testing procedures and the poor attendance of women at screening activities [5, 6]. Therefore, the development of newer testing options, with requirements that are less difficult to achieve, is contributing to increasing cervical screening and the potential reduction of cervical cancer cases in some developing countries [8–15]. These testing options include, visual inspection of the cervix after the application of either acetic acid or Lugol’s iodine (VIA or VILI), self-sampling based HPV testing and Liquid based cytology [12, 16, 17].

In order to reach out to women in different populations (both in developing and developed countries) with these testing options, different strategies have been developed either as standalone or to complement the traditional strategy of requesting women to report to a hospital. One of these new strategies include, providing incentives (mainly transportation) to encourage women to go to a health facility for self-specimen collection [18, 19]. Other strategy is to reach women with specimen collection kit at home (door-to-door approach), by sending the specimen collection kits to the women and get it returned by the women through the regular postal mail system. Response rates to this door-to-door approaches have ranged between 19.6% and 39.0% [20–23]. However, improvement of the door-to-door approach resulted in higher response rates. This improvement involved health personnel delivering the self-collection kits at the doors of the women, providing them information on cervical cancer and on how to use the kits. Thereafter, the women were asked to return the collected specimen to a hospital. In a study that compared this improved door-to-door approach to Pap testing at a hospital, more women (64.7%) opted for self-sampling and that more of the women in the self-sampling group (80.5%) returned their samples to the health facilities for testing. The 35.3% who opted for Pap testing had only 40.5% of them reporting to the hospital for Pap testing [18].

Ghana like other developing countries, is experiencing a number of challenges in respect of cervical cancer prevention. These challenges include a low screening coverage, estimated to be between 2.2% and 8.8%, the absence of a national cervical cancer screening programme, lack of a national guideline for cervical cancer screening and extremely localised opportunistic cervical screening service, which are centred mainly in two cities, Accra and Kumasi [24, 25]. Additionally, the implementation of these door-to-door approaches in Ghana and other developing countries will be greatly limited by the following; the provision of incentives cannot be supported by the economy in a nationwide cervical screening programme; the need for a high literacy level to ensure women are able to read, understand and follow the instructions on the self-collection procedures on their own, the need for repeated visits to homes in order to meet women and provide the information as to how to use the collection kits, absence of a functional and efficient postal system that can transport biological samples, the time needed by the women to return the collected specimen to a hospital and the absence of a good address system for follow-ups. This supposition is informed by the fact that the implementation of these door-to-door strategies in other countries have reported different rates of success [20–23]. The following limitations were experienced in those studies; lack of time to go to hospitals, discomfort with performing some procedures on their own, inconvenience in respect of daily activities, socio-cultural objections, structural and intra/interpersonal factors, unwillingness and inability to travel to health facilities [7, 18, 26].

Therefore, there is the need to develop a population specific strategies which will help overcome these limitations in Ghana, and other developing countries with similar sociocultural and socioeconomic settings. One such population specific strategy (an improved door-to-door approach with self-specimen collection for HPV testing) requires health workers to deliver the self-collection kits at the doors of the women, provide information on cervical cancer, teach them how to use the kits and ask the women to collect and return the specimen to them immediately. The few studies that have reported findings with this improved strategy have shown a high coverage rate; 97.1% coverage in Uganda [27], 85.8% coverage in Argentina [28] and 99.2% coverage in another study Uganda [8]. The starting aim of the study was to determine the response rate of women to a hospital-based cervical cancer screening activity, however, significant developments occurred during the study and lessons were learnt. In this report, we present the lessons learnt and the development of a different improvement to the door-to-door strategy, and comparison with the initial hospital-based specimen collection strategy. This improvement was a result of interactions we had with women in the communities following an initial low response rate with the hospital-based specimen collection strategy.

## Methods

### Study design

In this cross sectional study that took place between March 2012 and March 2013, one of five sub-districts of the Lower Manya Krobo District (Akuse sub-district) was randomly selected and all its communities included in the study. Three strategies for reporting for specimen collected were compared.

### Study location

The Lower Manya Krobo District of the Eastern Region was the district with highest STI prevalence and therefore was selected for this study. Subsequently, the Akuse sub-district of the Lower Manya Krobo District was randomly selected, among the 5 sub-districts, by random number selection using the Ms. Excel software. The Akuse sub-district is made-up of 18 communities, 12 of which are located closely (centred at the coordinates 6″06′02.43 N and 0″7′16.87E) while the other 6 are between 3 km and 5 km west of the 12 communities. The sub-district has a Government District Hospital with some of the communities having a Community Based Health Planning and Services (CHPS) Centre. There were four common Ghanaian languages spoken within the 18 communities of the sub-district; these were Hausa, Ewe, Ga-Adangbe and Twi. The study questionnaire was pre-tested in the Atua community of the Odumase sub-district.

### Study population

At the time of the design of this study (October, 2010), the estimated population of the Akuse sub-district for the year 2011 was 8887, of which 29.2% (2595) were females between the ages of 15 and 65 years [29]. A target of 410 women for the study was estimated based on the random sample size equations with an estimated HPV prevalence of 26.3%, degree of accuracy of 0.065, a design factor of 1.2, and an anticipated response rate of 60.0%, all of these were based on data from a population based cervical screening studies in neighboring West African countries [30]. Based on the population of women between the ages of 15 years and 65 years in each community, the number of women targeted for the study (410 participants) was distributed among 17 communities by the probability proportional to size.

Healthy women (self-report) between the ages of 15 and 65 years who were willing to provide cervical specimen by either self-collection or health-personnel specimen collection or both were eligible to participate in this study. Women who were pregnant (self-report) at the time of sample collection or had given birth in less than four months or had undergone hysterectomy or cervical conisation were excluded from the study. Women who had never had sexual intercourse were also excluded from the study.

### Community entry and engagement

The Akuse Government Hospital was determined to be the most influential institution within the sub-district through which the community could be successfully engaged. As such, with the assistance of the hospital administration, a meeting involving the religious leaders of the orthodox churches, researchers and the hospital administration took place. A community education programme was planned and presentations on cervical cancer and the study were made in two churches during the Christian Home Week Celebration, (during this celebration, members of all the orthodox churches in the sub-district meet in one of the churches for specific activities). Announcements were made regularly at all the orthodox churches regarding the study and its presence in the communities. Another presentation was made mid-way through the field work in a different church during a women’s group celebration. The leaders of the Muslim Community were separately contacted with the assistance of the hospital and during a Friday Muslim Prayer Service. The community members were informed of the study and issues relating to cervical cancer were discussed.

Each of the communities in the sub-district was entered by contacting the traditional leaders of the community, with the assistance of the Nurses of the Public Health Unit, who were well known to the leaders. The community members were reached by the traditional/local system of announcement and the assistance of the Community Health Volunteers within those communities. The participants were subsequently recruited by home visits. The Public Health Nurses and the Community Health Volunteers engaged the communities and participants continuously during their regular community outreach programmes, during which they explained the study and provided information about cervical cancer as well as taking feedbacks from the study team to the members of the community and participants. This study completely avoided the involvement of the political leaders and/or members of the local government system because of the existence of some political disputes to ensure freedom and willingness of participation.

### Sampling procedure

In identifying women by a house survey in each of the communities; the major roads through each community were identified by the use of the Google Map application and grouped in order of direction, from one end of a community to the other. Starting from the first randomly selected road, every third house to the right and left of the road/path were visited. Women in each house were provided information on cervical cancer and screening methods available and were encouraged to go for screening. Those who met the inclusion criteria were invited to participate in the study after obtaining informed consent. This was repeated for each road or path until the desired sample size was attained for each community.

### Compliance with ethical standards

The study was conducted in accordance with the ethical standards as laid down in the 1964 Declaration of Helsinki and its later amendments and comparable ethical standards. The participants either read and signed an informed consent form (in English) or it was translated to a local language and explained to them with the assistance of a witness; the explanation in each of the four local languages (Hausa, Ewe, Ga-Adangbe and Twi) was performed by one particular person throughout the study. The informed consent included the main research goals, sample collection procedures, potential benefits and harms, and privacy and confidentiality. The name and mobile phone number(s) (personal or that of another family member) of the women were obtained and each was recorded at separate places alongside a unique identification code, after informed consent had been obtained. The study was approved as a part of a bigger study by the Ethics Review Committee of the Ghana Health Service (ID No GHS-ERC: 06/11/10).

### Questionnaire administration

Two trained Public Health Nurses assisted the study participants, by a one-on-one interview to complete a study questionnaire, in such a manner as to ensure that no other person heard the participant and the Nurse. Information on the socio-demographic characteristics, as well as sexual and reproductive behaviour history, menstrual factors, use of oral contraception, and history of sexually transmitted infections and cervical cancer screening history were obtained (data not presented in this report).

### Strategies for specimen collection

#### Strategy one: Hospital-based sample collection

**Fig. 1:**
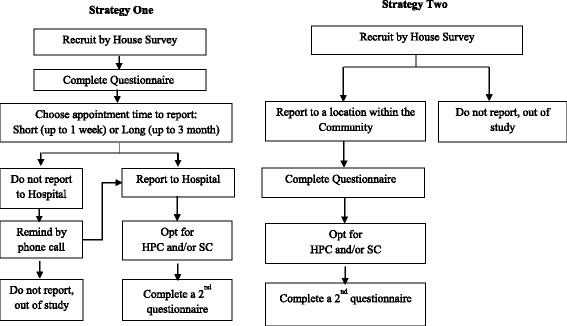
An illustration of the designed strategies for reporting for specimen collection

The consenting women who completed the questionnaire during the house survey were given the option to choose a convenient date; either within a week (short appointment) or any time after a week but not more than 3 months (long appointment) within which they were going to report at the hospital for both self-specimen collection and health personnel specimen collection. Those who did not report to the hospital, for more than two weeks after the date they opted for had elapsed, were called on the mobile phone number(s) they had provided until they were reached once and reminded. A new date was then arranged for them to visit the hospital for sample collection (Fig. 1). Although the above was the original strategy designed for the collection of specimen during this study, it was observed that some of the recruited women, who reported for specimen collection at the hospital, came along with other women who they had encouraged on their own initiative. This category of women was referred to as *Peer-Recruited* and for each of them, informed consent was obtained as described above and completion of study questionnaire was done at the hospital as described above, before specimens were collected.

#### Strategy two: Community-based sample collection

Although strategy one (described above) was the original strategy designed for this study, a consistent monitoring of the response to that strategy during the study revealed a very low response rate. In order to improve this low response rate, this second strategy evolved that was based on the difficulties identified during discussions with women, community health workers and some members of the communities (including participants were called as part of strategy one to reminded them of their appointment).

This second strategy involved identifying, a location within a community where a room was available for the women to be attended to in privacy. This location was mostly central, well known and convenient to quickly reach from all other parts of the community. Women recruited by the house survey were invited to participate by going to the selected location at any time between 9:00 am and 7:00 pm. Women who reported were invited to participate in the study after taking them through the consenting process as described above. Consenting women who met the inclusion criteria were assisted to complete the study questionnaire as described above.

### Specimen collection

The participants were given the option to perform both self-collection and health personnel collection, both with the hospital and the community collection strategies. The process for self-collection of cervical swabs (self-specimen collection) was described to the participants by a one-on-one interaction and with the aid of pictures/illustrations. The self-specimen collection was done by the participants alone in a separate room and with the illustrations of how to perform the process at hand. The vaginal examination and health personnel specimen collection was performed with only the health personnel (Medical Officer or Nurse) and the participant behind a screen in a room. After samples collection, the participants completed a second questionnaire about their experience and future preferences in respect of the collection of specimen.

### Variables and statistical analysis

Data on the participants’ demographic characteristics, as well as sexual and reproductive history (data not shown in this publication) were collected and assessed as predictors of the major outcomes of the study. These major outcome variables which included the following; the overall and individual response rates for each of the reporting strategies employed, the proportions of participants who performed self, health-personnel or both specimen collection methods, the proportions of participants who post-performance preferred self, health-personnel or any of specimen collection methods, reasons for choose of performed method and preferences, were described as proportions of their respective totals. Chi-square analysis was used to assess the associations between demographic characteristics and the reporting for specimen collection for each of the three strategies as well as the association with the preference for specimen collection methods.

## Results

### Reporting for specimen collection

**Fig. 2:**
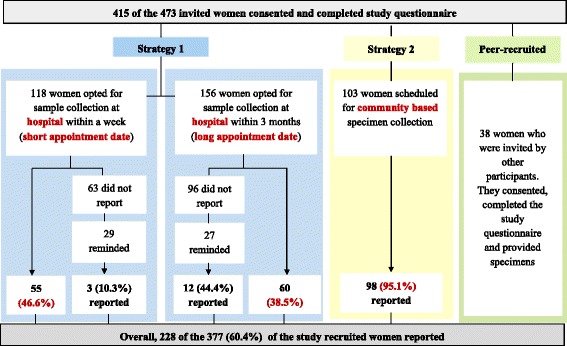
Participant enrolment and response to the different strategies for specimen collection

Of the 473 eligible women invited to participate in this study, 415 consented and completed the study questionnaire. Of these, 377 participants were invited by the researchers while 38 were invited by some of the women who reported for specimen collection (these women were designated *peer-recruited*). Since the women invited by their peers, did not fit in any of the reporting strategies, these women were excluded from the analysis of the reporting rates, in order to avoid obtaining a biased estimate of the overall reporting rate (Fig. 2).

#### Strategy one (hospital based)

Of the 377 consenting participants, 274 were invited to participate using reporting strategy one. Among these, 118 of them opted to report within a week (Short appointment time), while 156 opted to report after a week but not more than 3 months (Long appointment time), for specimen collection at the hospital (Fig. 2). Among the 156 participants who opted for the long appointment time, 60 (38.5%) reported for specimen collection at the hospital within the stated period. Of the remaining 96 participants who did not report, 27 were reached by a phone call, reminded of their participation and were allowed to set a new appointment date. However, only, 12 of these 27 (44.4%) called participants reported on the new dates for specimen collection at the hospital. On the other hand, among the 118 participants who opted for the short appointment time to report to the hospital, 55 (46.6%) reported within the time they opted for. However, of the remaining 63 participants who did not report, 29 were reached by a phone call, reminded of their participation and allowed to fix a new appointment date. Interestingly, of these 29 called participants, only 2 (10.3%) reported to the hospital for specimen collection by the new appointment date.

#### Strategy two (community-based)

In respect of the strategy that was developed on the field (reporting strategy two), 103 women were invited to participate in this study and 98 (95.1%) of these women reported for specimen collection at the location within their respective communities and within the expect time (a week) they indicated to report (Fig. 2).

#### Overall reporting rate

Overall, 228 of the 377 participants recruited by the research team (excluding the 38 peer-recruited participants) reported for specimen collection, resulting in a reporting rate of 60.4% for this study. A comparison of the response rates for the three reporting strategies (long appointment time to report to the hospital, short appointment time to report to the hospital and report at a location within community), showed a statistically significant difference (*p = 0.0001*) and an LSD multiple comparison (post hoc analysis) indicated that all the pairwise comparisons of response rates were significantly different. The order therefore, from highest to lowest was, reporting at a location within a community (95.1%) > short appointment time to report (46.6%) > long appointment time to report (38.5%).

### Demographic characteristics

Data of the 38 peer-recruited women were included in the analysis of the demographic characteristics because they were similar to those of the women who were recruited by the research team (Table 1). The ages of the 415 participants ranged between 15 years and 70 years with a median of 31.0 years and an interquartile range (IQR) of 16.0 years; Shapiro-Wilk test of normality showed a significant difference, *p = 0.0001*. The age group with the highest proportion of the participants was 25–29 years (*n* = 97; 23.5%), while that with the lowest proportion was the age groups 60 years and older (*n* = 10; 2.4%). A majority of the participants were Christians (90.5%: *n* = 373), and more than half (63.1%; *n* = 257) of them were married. Among the women who were married, 79.4% (197 of 248) were in monogamous marriages, while 14.9% (*n* = 37) were in polygamous marriages. The commonest educational level 47.4% was the Junior Secondary level. Additionally, 6.2% of the women were unemployed, and the occupations of those who were employed were categorised into four groups. Traders (42.8%) and skilled workers (23.1%) formed the majority of the participants. The others were either in formal employment (16.4%) or in Agro-business (11.4%).

**Table 1:** Stratified and overall distributions of the demographic characteristics of the recruited participants

	Sub-categories	Reporting strategy, *n* (%)				All women
		Community based	Peer Recruited	Long appointment	Short appointment	
Age	15–19	6 (5.8)	2 (5.6)	12 (7.7)	6 (5.1)	26 (6.3)
	20–24	18 (17.5)	2 (5.6)	26 (16.8)	19 (16.1)	65 (15.8)
	25–29	28 (27.2)	8 (22.2)	36 (23.2)	25 (21.2)	97 (23.5)
	30–34	6 (5.8)	3 (8.3)	20 (12.9)	17 (14.4)	46 (11.2)
	35–39	12 (11.7)	7 (19.4)	15 (9.7)	18 (15.3)	52 (12.6)
	40–44	8 (7.8)	8 (22.2)	18 (11.6)	17 (14.4)	51 (12.4)
	45–49	6 (5.8)	2 (5.6)	15 (9.7)	7 (5.9)	30 (7.3)
	50–54	8 (7.8)	1 (2.8)	10 (6.5)	3 (2.5)	22 (5.3)
	55–59	4 (3.9)	3 (8.3)	2 (1.3)	4 (3.4)	13 (3.2)
	60 Or Older	7 (6.8)^a^	0 (0.0)	1 (0.6)	2 (1.7)	10 (2.4)
	Total	103	36	155	118	412 (100)
Religion	Christian	103 (100)	36_,_^(^100)	129 (82.7)^a^	105 (89.7)	373 (90.5)
	Muslim	0 (0.0)	0 (0.0)	25 (16.0)^a, b^	10 (8.5)^b^	35 (8.5)
	Other	0 (0.0)	0 (0.0)	2 (1.3)	2 (1.7)	4 (1.0)
	Total	103	36	156	117	412 (100)
Marital Status	Unmarried	38 (38.0)	10 (28.6)	49 (31.8)	53 (44.9)	150 (36.9)
	Married	62 (62.0)	25 (71.4)	105 (68.2)	65 (55.1)	257 (63.1)
	Total	100	35	154	118	407 (100)
Educational Status	No Formal	21 (20.8)	6 (16.7)	30 (19.5)	11 (9.3)	68 (16.6)
	Primary	26 (25.7)	5 (13.9)	30 (19.5)	16 (13.6)	77 (18.8)
	Junior Secondary	39 (38.6)	14_,_(38.9)	69 (44.8)^a^	72 (61.0)^a^	194 (47.4)
	Senior Secondary	9 (8.9)	6 (16.7)	18 (11.7)	15 (12.7)	48 (11.7)
	Post-Secondary	6 (5.9)	5 (13.9)	7 (4.5)	4 (3.4)	22 (5.4)
	Total	101	36	154	118	409 (100)
Occupation	Unemployed	12 (12.0)	0 (0.0)	7 (4.7)	6 (5.2)	25 (6.2)
	Formally employed	18 (18.0)	9 (25.0)	23 (15.3)	16 (13.8)	66 (16.4)
	Skilled Worker	23 (23.0)	8 (22.2)	25 (16.7)	37 (31.9)	93 (23.1)
	Trader	34 (34.0)	16 (44.4)	75 (50.0)	47 (40.5)	172 (42.8)
	Agro-worker	13 (13.0)	3 (8.3)	20 (13.3)	10 (8.6)	46 (11.4)
	Total	100	36	150	116	402 (100.0)

^a^Denotes for each sub-category’s column proportions for a reporting strategy that differ significantly from the others of the same demographic character, at the 95% confidence level

^b^The difference in religious characteristics that showed Muslim in only two of the four strategies was primary due to the fact that there was a major Muslim community that was only approached at the early stage of the study with the Hospital long and short duration based strategies

### Participants’ characteristics that may have influenced Reponses for each strategies

It was determined among the participants approached with the community-based strategy as well as those approached with the hospital-based long appointment time strategy that their reporting for specimen collection was not associated with any of their demographic characteristics (Table 2). However, among the women who opted for the short appointment time, age (categorised/grouped) of the participants was significantly associated (*p = 0.037*) with reporting for specimen collection (Table 2). Women between the ages of 20 and 39 years reported the most (Additional file 1: Tables S1 – S3).

**Table 2:** Association between participants’ characteristics and reporting for each strategy of specimen collection

Characteristics	χ^2^ (*p* value)		
	Community	Hospital (Long)	Hospital (Short)
Age	6.097 (0.730)	13.657 (0.135)	17.863 (0.037)^a^
Marital status	(0.633)^‑^	2.124 (0.145)	2.146 (0.143)
Religion	64.4 (0.001)	2.672 (0.263)	3.331 (0.189)
Educational status	9.205 (0.056)	4.576 (0.334)	2.406 (0.662)
Occupation	4.173 ^(^0.383)	5.717 (0.221)	4.046 (0.400)

^a^Fisher exact

### Performance of specimen collection method

Overall, 253 participants performed specimen collection (Table 3) and 226 (89.3%) of these participants provided specimen by both self-collection (SC) and health-personnel collection (HPC). The 7 (2.8%) participants who provided only health personnel collected specimen did so, according to them, mainly because of blindness and the fear of hurting themselves with the self-specimen collection brush. On the other hand, 20 (7.9%) participants provided only self-collected specimen and this was, according to them, mainly because they did not want the insertion of the speculum. Some of the women were just afraid of the speculum while others resisted the speculum insertion; the labels of SC specimens of 2 women were lost while the labels of the HPC specimens of other two women were also lost. Of the participants who provided SC specimen (20 + 226), 227 (92.3%) stated their opinion about the self-specimen collection brush (Rover Viba-Brush Vaginal Sampler). Among these, 205 (90.3%) were of the opinion that the Rover Viba-Brush Vaginal Sampler was easy to use, while 22 (9.7%) of them stated that it was difficult to use.

**Table 3:** Performance and Preferences for Self and Health personnel collected specimen

Preference	SC only	HPC only	Both^a^/Any^b^	Total
Performance	20 (7.9%)	7 (2.8%)	226 (89.3%)	253
Post-performance preference	51 (22.6%)	127 (56.2%)	49 (21.7%)	226

^a^For performance

^b^For post-performance preference

### Post-performance preferences for specimen collection method

Slightly more than half of the 226 participants (56.2%; *n* = 127) who performed both specimen collection stated that they preferred the health personnel specimen collection. While less than one-fourth (22.6%; *n* = 51) of them stated that they preferred the self-specimen collection. The others did not have any particular preference (21.7%; *n* = 49) (Table 3). The reasons for the post-performance preference for self-specimen collection as stated by the participants included the experience of slight pain and/or fear experienced during the health personnel specimen collection. Other reasons for this post-performance preference were the privacy it provided and how simple it was to perform (Table 4). On the other hand, the reasons for the post-performance preference for health personnel specimen collection (HPC) as stated by the participants were that the HPC was most likely better performed than the self-specimen collection (SC), because the Nurses and Medical Officers were professionals, experienced and knew what they were about (Table 5). Also, some of the participants stated that since they had no knowledge of cervical cancer screening, the health personnel specimen collection was preferable. Some of the participants were not confident they had performed the self-specimen collection as expected. It was also determined that the post-performance preference of the participants was not significantly associated with their demographic characteristics (*χ* ^*2*^ *of 15.54; p > 0.05*) as well as with their opinion about the use of the Rover Viba-brush vaginal sampler (Additional file 1: Table S4).

**Table 4:** Reasons for the post-performance preference for self-specimen collection

Reasons	Frequency	Percentage
Health personnel sample collection was with slight pain and /or fear	35	68.6
Issues of privacy	6	11.8
Self-specimen collection was simple and easier to perform	6	11.8
Can’t explain	1	2.0
Stated no reason	3	5.9
Total	51	100.0

**Table 5:** Reasons for the post-performance preference for health personnel specimen collection

Reasons	Frequency	Percentage
The health personnel would have done theirs better because they were professionals, experienced and know what they were about	85	66.9
The participants had no idea about cervical screening	16	12.6
The participants were not confident they performed the self-specimen collection correctly.	14	11.0
In order to obtain good and/or the right results	2	1.4
The health personnel would find another problem or unusual occurrences if any around the sampling area	2	1.4
The pap smear was not difficult and there was no pain	1	0.7
Participant was afraid she was going to hurt herself with the self-specimen collection device	1	0.7
The participant stated that the self-sampling device is not user-friendly	1	0.7
The nurse could see what she was doing, but the participant did not see what she did during self-specimen.	1	0.7
Had no problem with the health personnel examinations and sample collection	1	0.7
Gave no reason	6	4.7
Total	127	100.0

## Discussion

The Akuse sub-district, Ghana, was suspected to be a community at high risk for HPV, due to the high reported rate of STI by hospitals within the sub-district as such was chosen for an HPV and cervical lesion prevalence study. The data presented here forms part of that bigger study. The attained overall response rate of 60.4% was only slightly higher than the anticipated rate of 60.0%, which used in the determination of the sample size. Additionally, compared to the varying response or coverage rates reported by recent studies of cervical screening in different global regions, which range from 48% to 88% [20–23], the overall response rate attained in this study was considered relatively high, since this was achieve without the provision of incentives to the participants. In respect of the response rate to the individual strategies employed in this study, the high response rate of 96.1% (Fig. 2) attained by the community-based specimen collection strategy was interesting. This was most likely due to the fact that the participants were able to easily reach the collection centres, which were located within their respective communities, and that they were allowed to report later in the day, particularly between 3:00 pm and 7:00 pm, when most of them were or had returned from their daily activities. These probably made it easier for the women to participate in the study without it interfering with their regular daily activities. This finding implies this community-based strategy is an appreciable improvement over most of the widely reported forms/types/versions of the door-to-door strategies for cervical screening. The older versions of the door-to-door strategy have reported response rates ranging between 8.7% and 39.1% [31], and the more recent version have reported response rates of between 80.0% and 99.0% [8, 27, 28]. Additionally, the high rates reported in this study was so due to the fact that the strategy employed herein avoided some of the major difficulties of the widely reported version of the door-to-door strategy. That is, our strategy provided assistance in understanding the self-collection process, avoided the need to return the specimen either by post, by taking it to the hospital or for a nurse to come back to the home of the women for the specimen, avoided the possibility of the women postponing and possibly forgetting to perform the specimen collection, and avoided the need for a health worker to keep visiting the house of a women until the woman is meet and invited to participate. It is also worth noting that most of the participants reported for specimen collection within a week after the house survey. Together, these suggest a high potential for this community-based specimen collection strategy as a stand-alone strategy at improving response to cervical cancer screening services in similar settings of other developing countries.

On the other hand, the findings of the hospital-based specimen collection strategies, indicate that requesting women to report to a hospital within a short time (short appointment time after a home based recruitment process) is potentially good for recording a substantial cervical screening coverage (reporting rate of 46.6%), if a hospital based strategy is the only option for a community. This is because the response rate for the longer appointment time to report to the hospital, attained a lower response rate of 38.5%. This response rate was lower than the 60.0% observed in a similar cervical cancer screening study in USA, where a home-based recruitment was followed with a hospital-based specimen collection and the provision of incentives [18, 32]. Furthermore, reminding non-responding women with a phone call may only contribute marginally to improving response rate or coverage because of the very low and moderate responses (10.3% and 44.4%) of the non-responders who were called for this study. However, this might be the only effective means of reminding women in Ghana, since the other available means of re-inviting non-responders for specimen collection at a hospital (sending letters) are less likely to be effectively operational in Ghana.

After reporting for specimen collection during a cervical cancer screening programme, the important issue to consider is the performance and preference for a specimen collection method. For this study, a high acceptance of both specimen collection methods used (self and health personnel) was evident by the high proportion of the women (89.3%; *n* = 226), who opted for both specimen collection methods. Additionally, the overall proportion of women who performed self-collection, 97.2% (246 of 253) and health-personnel specimen collection, 92.1% (233 of 253) in our study were slightly higher than those reported by a study in Cameroon; where 29.0% of the women performed self-collection and 62.0% performed health personnel collection [33] as well as in a study in the USA where 80.5% of the women performed self-specimen and 40.5% of them performed health personnel specimen collection [18]. However, the performance rates for this study were similar to the 98.0% who performed self-specimen and 87.0% performed health personnel specimen collection in a Mexican study [34]. Despite these high performance rates for both methods, the 226 participants who performed both specimen collections clearly indicated a higher post-performance preference for health personnel specimen collection (56.2%) and a lower post-performance preference for self-specimen collection (22.6%). These variation in preference may suggest that the participants’ expectation before they perform the specimen collection were not what they experience when they performed the specimen collection. These preference rates were both lower than those reported in a study of urban women who reported post-performance preference of 68.0% for health personnel collection and 32.0% for self-collection, after they had experienced both methods [35]. However, it must be noted and as indicated in a review by Schmeink et al., [36] that some studies report post-performance preference while other report only pre-performance preference [18].

Most of the reasons stated by the participants of this study for their preferences (Tables 4 and 5) have been reported by participants of other studies who preferred health personnel specimen collection [26, 33, 36–39]. Similarly, the participants who preferred self-specimen collection gave similar reasons for their preference as has been reported in some other studies by participants who preferred self-specimen collection [21, 26, 36]. However, it was interesting to note that some of the participants were not confident in their performance of the self-specimen collection although they stated that it was simple and easy for them to use. This inconsistency between performance and preference suggests the need for educating participants on the peculiarity and usefulness of self-specimen collection in order to improve its acceptability as part of cervical cancer screening activities. It was also determined that the preferences for the collection methods were not associated with and therefore were not influenced by the demographics of the participants (*p* values >0.05) as has been reported by most studies [22, 35, 39].

This study was limited by the fact the community based strategy evolved on the field, and therefore only 4 of the 17 communities that had not yet been involved in the study were reached with this strategy and as such the study was not randomised enough. By the nature of this strategy (particularly the fact that there was more time to report within a day and that the completion of questionnaire was at the collection point but not at home during recruitment), the level of motivation for the women in these 4 communities were different from those who were to report at the hospital. Therefore, differences in performance between this and the other approach are mainly suggestive of their relative potential influence on cervical cancer screening activities in Ghana and other developing countries with similar settings.

## Conclusions

Based on the lessons learnt during our study, we are of the view that cervical cancer screening programs will have a better coverage rate if specimen collection points are set up within communities where women will be invited to go for screening rather than going to homes and asking women to report to hospitals for screening. The findings of this study has shown good potential for increasing women participation in cervical cancer screening in Ghana and sub-Saharan Africa countries with similar community settings. However, if the cervical screening services must be hospital-based, then inviting women to report within a short duration after recruitment holds good potential for increasing women’s participation. The findings of this study points to the need for further and larger feasibility or pilot studies to determine the effectiveness and efficiency of this community-based specimen collection strategy for cervical screening services in low to middle income/developing countries.

## Additional file

Additional file 1: Table S1. Association between participants’ characteristics and reporting for community-based reporting strategy of specimen collection. **Table S2.** Association between participants’ characteristics and reporting for hospital long appointment reporting strategy of specimen collection. **Table S3.** Association between participants’ characteristics and reporting for hospital short appointment reporting strategy of specimen collection. **Table S4.** Association between participants’ characteristics and post-performance preference for specimen collection methods. (DOCX 27 kb)

## Abbreviations

CHPS: Community Based Health Planning and Services
HPC: Health-personnel collection
IQR: Interquartile range
SC: Self-collection
VIA: Visual inspection with acetic acid
VILI: Visual inspection with Lugol’s iodine
