# Supplementary material for: A tailored within-community specimen collection strategy increased uptake of cervical cancer screening in a cross-sectional study in Ghana
Source: BMC Public Health. 2017 Aug 1;18:80. doi: 10.1186/s12889-017-4631-y (PMC5540566; doi:10.1186/s12889-017-4631-y)
Supplement: Additional file 1: Table S1. — Association between participants’ characteristics and reporting for community-based reporting strategy of specimen collection. Table S2. Association between participants’ characteristics and reporting for hospital long appointment reporting strategy of specimen collection. Table S3. Association between participants’ characteristics and reporting for hospital short appointment reporting strategy of specimen collection. Table S4. Association between participants’ characteristics and post-performance preference for specimen collection methods. (DOCX 27 kb) [file 12889_2017_4631_MOESM1_ESM.docx]

**Community-Based Specimen Collection for Improved Cervical Cancer Screening in a Low Income Community: A Cross-Sectional Study in Ghana**

Adolf K Awua^1,2,§^, Edwin K Wiredu^4^, Edwin A Afari^1^, Ahmad S Tijani^6^, Gabriel Djanmah^6^ Richard M K Adanu^3^.

**Affiliations**

^1^Department of Epidemiology and Disease Control, School of Public Health, University of Ghana.

^2^Cellular and Clinical Research Centre, Radiological and Medical Sciences Research Institute, GAEC, Ghana.

^3^Population, Family and Reproductive Health, School of Public Health, University of Ghana

^4^University of Health and Allied Sciences, Ghana.

^5^Department of Pathology, School of Biomedical and Allied Health Science, College of Health Sciences, University of Ghana, Korle-Bu, Ghana.

^6^Akuse Government Hospital, Ghana Health Service.

**Supplementary Tables**

Table S1: Association between participants’ characteristics and reporting for community-based reporting strategy of specimen collection

| **Variables** | **Categories** | **Community-based approach for specimen collection, n (%)** | | **Total, n(%)^#^** | **χ^2^**  **(p value)** |
| --- | --- | --- | --- | --- | --- |
|  |  | **Did not report^*^** | **Reported^*^** |  |  |
| Age (categorised) | 15 - 19 | 0 (0.0) | 6 (100.0) | 6 (5.8) | 6.097 (0.730) |
|  | 20 - 24 | 2 (11.1) | 16 (88.9) | 18 (17.5) |  |
|  | 25 - 29 | 1 (3.6) | 27 (96.4) | 28 (27.2) |  |
|  | 30 - 34 | 0 (0.0) | 6 (100.0) | 6 (5.8) |  |
|  | 35 - 39 | 0 (0.0) | 12 (100.0) | 12 (11.7) |  |
|  | 40 - 44 | 1 (12.5) | 7 (87.5) | 8 (7.8) |  |
|  | 45 - 49 | 0 (0.0) | 6 (100.0) | 6 (5.8) |  |
|  | 50 - 54 | 0 (0.0) | 8 (100.0) | 8 (7.8) |  |
|  | 55 - 59 | 0 (0.0) | 4 (100.0) | 4 (3.9) |  |
|  | 60 or older | 0 (0.0) | 7 (100.0) | 7 (6.8) |  |
|  | Total | 4 | 99 | 103 |  |
| Educational status | No formal education | 0 (0.0) | 21 (100.0) | 21 (20.8) | 9.205 (0.056) |
|  | Primary | 1 (3.8) | 25 (96.2) | 26 (25.7) |  |
|  | Junior secondary | 1 (2.6) | 38 (97.4) | 39 (38.6) |  |
|  | Senior secondary | 2 (22.2) | 7 (77.8) | 9 (8.9) |  |
|  | Post-Secondary | 0 (0.0) | 6 (100.0) | 6 (5.9) |  |
|  | Total | 4 | 97 | 101 |  |
| Occupation | Unemployed | 12 (100.0) | 0 (0.0) | 12 (12.0) | 4.173^b^  (0.383) |
|  | Formal employment | 17 (94.4) | 1 (5.6) | 18 (18.0) |  |
|  | Skilled worker | 23 (100.0) | 0 (0.0) | 23 (23.0) |  |
|  | Trader | 31 (91.2) | 3 (8.80) | 34 (34.0) |  |
|  | Agro-worker | 13 (100.0) | 0 (0.0) | 13 (13.) |  |
|  | Total | 96 | 4 | 100 |  |
| Marital status | Unmarried | 2 (5.3) | 36 (94.7) | 38 (38.0) | Fisher exact (0.633) |
|  | Married | 2 (3.2) | 60 (96.8) | 62 (62.0) |  |
|  | Total | 4 | 96 | 100 |  |
| Religion | Christian | 4 (3.8) | 99 (96.2) | 103 (100) | NA |
|  | Muslim | 0 (0.0) | 0 (0.0) | 0 (0.0) |  |
|  | Other | 0 (0.0) | 0 (0.0) | 0 (0.0) |  |
|  | Total | 4 | 99 | 103 |  |
| ^* % are of the row totals # % are of column totals^ | | | | | |

Table S2: Association between participants’ characteristics and reporting for hospital long appointment reporting strategy of specimen collection

| **Variables** | **Categories** | **Long appointment approach for specimen collection at the hospital, n(%)** | | **Total, n(%)^#^** | **χ^2^**  **(p value)** |
| --- | --- | --- | --- | --- | --- |
|  |  | **Did not report^*^** | **Reported^*^** |  |  |
| Age (categorised) | 15 - 19 | 3 (33.3) | 6 (66.7) | 9 (7.0) | 13.657 (0.135) |
|  | 20 - 24 | 6 (41.8) | 16 (59.2) | 22 (17.2) |  |
|  | 25 - 29 | 9 (32.1) | 19 (67.8) | 28 (21.9) |  |
|  | 30 - 34 | 5 (29.4) | 12 (70.6) | 17 (13.3) |  |
|  | 35 - 39 | 6 (46.2) | 7 (53.8) | 13 (10.2) |  |
|  | 40 - 44 | 8 (61.6)) | 5 (38.4) | 13 (10.2) |  |
|  | 45 - 49 | 3 (23.1) | 10 (76.9) | 13 (10.2) |  |
|  | 50 - 54 | 6 (60.0) | 4 (40.0) | 10 (7.8) |  |
|  | 55 - 59 | 2 (100.0) | 0 (0.0) | 2 (1.6) |  |
|  | 60 or older | 1 (100.0) | 0 (0.0) | 1 (0.8) |  |
|  | Total | 49 | 79 | 128 |  |
| Educational status | No formal education | 7 (35.0) | 21 (75.0) | 28 (22.0) | 4.576 (0.334) |
|  | Primary | 13 (49.2) | 14 (51.8) | 27 (21.3) |  |
|  | Junior secondary | 23 (41.4) | 34 (59.6) | 57 (44.9) |  |
|  | Senior secondary | 5 (50.0) | 5 (50.0) | 10 (7.9) |  |
|  | Post-Secondary | 1 (20.0) | 4 (80.0) | 5 (3.9) |  |
|  | Total | 49 | 78 | 127 |  |
| Marital status | Unmarried | 11 (29.0) | 27 (71.0) | 38 (30.0) | 2.124 (0.145) |
|  | Married | 38 (42.7) | 51 (57.3) | 89 (70.0) |  |
|  | Total | 49 | 78 | 127 |  |
| Religion | Christian | 44 (40.7) | 64 (59.3) | 108 (85.0) | 2.672 (0.263) |
|  | Muslim | 5 (26.3) | 14 (73.7) | 19 (14.9) |  |
|  | Other | 0 | 2 (100.0) | 2 (1.6) |  |
|  | Total | 49 | 78 | 127 |  |
| Occupation | Unemployed | 3 (60.0) | 2 (40.0) | 5 (4.1) | 5.717 (0.221) |
|  | Formal employment | 5 (29.4) | 12 (70.6) | 17 (13.8) |  |
|  | Skilled worker | 7 (36.8) | 12 (63.2) | 19 (15.4) |  |
|  | Trader | 26 (41.3) | 37 (58.7) | 63 (51.2) |  |
|  | Agro-worker | 3 (15.8) | 16 (84.2) | 19 (15.4) |  |
|  | Total | 44 | 79 | 123 |  |
| ^* % are of the row totals # % are of column totals^ | | | | | |

Table S3: Association between participants’ characteristics and reporting for hospital short appointment reporting strategy of specimen collection

| **Variables** | **Categories** | **Short** **appointment approach for specimen collection at the hospital, n(%)** | | **Total, n(%)^#^** | **χ^2^**  **(p value)** |
| --- | --- | --- | --- | --- | --- |
|  |  | **Did not report^*^** | **Reported^*^** |  |  |
| Age (categorised) | 15 - 19 | 0 (0.00) | 6 (100.0) | 6 (6.7) | 17.863^d^  (0.037) |
|  | 20 - 24 | 10 (62.5) | 6 (38.5) | 16 (17.8) |  |
|  | 25 - 29 | 12 (66.7) | 6 (33.3) | 18 (20.0) |  |
|  | 30 - 34 | 11 (78.6) | 3 (22.4) | 14 (15.6) |  |
|  | 35 - 39 | 10 (71.4) | 4 (29.6) | 14 (15.6) |  |
|  | 40 - 44 | 6 (50.0) | 6 (50.0) | 12 (13.3) |  |
|  | 45 - 49 | 3 (50.0) | 3 (50.0) | 6 (6.7) |  |
|  | 50 - 54 | 1 (100.0) | 0 (0.00) | 1 (1.1) |  |
|  | 55 - 59 | 0 (0.00) | 2 (100.0) | 2 (2.2) |  |
|  | 60 or older | 0 (0.00) | 1 100.0) | 1 (1.1) |  |
|  | Total | 53 | 37 | 90 |  |
| Educational status | No formal education | 6 (54.5) | 5 (45.4) | 11 (12.2) | 2.406^d^  (0.662) |
|  | Primary | 8 (62.5) | 5 (38.5) | 13 (14.4) |  |
|  | Junior secondary | 31 (56.3) | 24 (43.6) | 55 (61.1) |  |
|  | Senior secondary | 5 (62.5) | 3 (38.5) | 8 (8.9) |  |
|  | Post-Secondary | 3 (100.0) | 0 (0.0) | 3 (3.30) |  |
|  | Total | 53 | 37 | 90 |  |
| Occupation | Unemployed | 3 (60.0) | 2 (40.06) | 5 (5.7) | 4.046 (0.400) |
|  | Formal employment | 5 (41.6) | 7 (59.4) | 12 (13.6) |  |
|  | Skilled worker | 20 (66.7) | 10 (33.3) | 30 (34.1) |  |
|  | Trader | 21 (63.6) | 12 (36.4) | 33 (37.5) |  |
|  | Agro-worker | 3 (38.5) | 5 (62.59) | 8 (9.1) |  |
|  | Total | 52 | 36 | 88 |  |
| Marital status | Unmarried | 19 (50.0) | 19 (50.0) | 38 (42.2) | 2.146e  (0.143) |
|  | Married | 34 (65.4) | 18 (34.6) | 52 (57.8) |  |
|  | Total | 53 | 37 | 90 |  |
| Religion | Christian | 50 (61.7) | 31 (38.3) | 81 (91.0) | 3.331^d^  (0.189) |
|  | Muslim | 3 (50.0) | 3 (50.0) | 6 (6.7) |  |
|  | Other | 0 | 2 (100.0) | 2 (2.2) |  |
|  | Total | 53 | 36 | 89 |  |
| ^* % are of the row totals # % are of column totals^ | | | | | |

Table S4: Association between participants’ characteristics and post-performance preference for specimen collection methods

| **Demographics** | **Categories** | **Post-performance Preference, n(%)^*^** | | | **Total, n(%)^#^** | **Pearson Chi-Square (p value)** |
| --- | --- | --- | --- | --- | --- | --- |
|  |  | **Self** | **Health personnel** | **Any** |  |  |
| Age (categorised) | 15 - 19 | 2 (18.2) | 8 (72.7) | 1 (9.1) | 11 (4.9) | 17.026 (0.521) |
|  | 20 - 24 | 6 (18.2) | 19 (57.6) | 8 (24.2) | 33 (14.6) |  |
|  | 25 - 29 | 17 (31.5) | 23 (42.6) | 14 (25.9) | 54 (23.9) |  |
|  | 30 - 34 | 5 (20.0) | 15 (60.0) | 5 (40.0) | 25 (11.1) |  |
|  | 35 - 39 | 5 (15.2) | 23 (69.7) | 5 (15.2) | 33 (14.6) |  |
|  | 40 - 44 | 5 (17.2) | 19 (65.5) | 5 (17.2) | 29 (12.8) |  |
|  | 45 - 49 | 5 (35.7) | 7 (50.0) | 2 (14.3) | 14 (6.2) |  |
|  | 50 - 54 | 2 (16.6) | 5 (41.7) | 5 (41.7) | 12 (5.3) |  |
|  | 55 - 59 | 2 (25.0) | 5 (62.5) | 1 (12.5) | 8 (3.5) |  |
|  | 60 or older | 2 (28.6) | 2 (28.6) | 3 (42.9) | 7 (3.1) |  |
|  | Total | 51 | 126 | 49 | 226 |  |
| Educational status | No formal education | 10 (27.0) | 18 (48.6) | 9 (24.3) | 37 (16.5) | 10.40 (0.238) |
|  | Primary | 7 (16.3) | 24 (55.8) | 12 (27.9) | 43 (19.2) |  |
|  | Junior secondary | 19 (18.4) | 61 (59.2) | 23 (22.3) | 103 (46.0) |  |
|  | Senior secondary | 8 (32.0) | 14 (56.0) | 3 (12,0) | 25 (11.2) |  |
|  | Post-Secondary | 7 (43.8) | 8 (50.0) | 1 (6.25) | 16 (7.10) |  |
|  | Total | 51 | 125 | 48 | 224 |  |
| Occupation | Unemployed | 3 (17.6) | 9 (52.9) | 5 (29.4) | 17 (7.7) | 15.54 (0.049) |
|  | Formal employment | 14 (38.9) | 19 (52.8) | 3 (8.3) | 36 (16.4) |  |
|  | Skilled worker | 16 (26.6) | 28 (46.6) | 16 (26.6) | 60 (27.3) |  |
|  | Trader | 15 (17.0) | 56 (63.6) | 17 (19.3) | 88 (40.0) |  |
|  | Agro-worker | 2 (10.5) | 10 (52.6) | 7 (36.8) | 19 (8.6) |  |
|  | Total | 50 | 122 | 48 | 220 |  |
| Marital status | Unmarried | 20 (28.2) | 37 (52.1) | 14 (19.7) | 71 (32.0) | 1.914 (0.384) |
|  | Married | 30 (198.7) | 87 (57.6) | 34 (22.5) | 151 (68.0) |  |
|  | Total | 50 | 124 | 48 | 222 |  |
| Religion | Christian | 51 (23.3) | 119 (54.3) | 49 (22.4) | 219 (96.9) | 5.733 (0.057) |
|  | Muslim | 0 (0.0) | 7 (100.0) | 0 (0.0) | 7 (3.1) |  |
|  | Other | 0(0.0) | 0(0.0) | 0(0.0) | 0(0.0) |  |
|  | Total | 51 | 126 | 49 | 226 |  |
| What Is Your Opinion About Use Of Sample Sampling Device | Easy | 49 (23.9) | 111 (54.1) | 45 (21.9) | 205 (90.3) | **3.297 (0.192)** |
|  | Difficult | 2 (9.1) | 16 (72.7) | 4 (18.2) | 22 (9.7) |  |
|  | Total | 51 | 127 | 49 | 227 |  |
| ^* % are of the row totals # % are of column totals^ | | | | | | |
